# DNA/RNA Methylation-Driven Coronary Microvascular Dysfunction: Emerging Pathogenic Mechanisms and Therapeutic Opportunities for Heart Failure in Diabetes

**DOI:** 10.1007/s10557-026-07907-1

**Published:** 2026-07-11

**Authors:** Ariana Bacelar-Jimenez, Ammar S. Alamareen, Vinuthna Vani Madishetti, Mohammad S. Samarah, Chris J. Watson, David J. Grieve, Karla M. O’Neill

**Affiliations:** Wellcome-Wolfson Institute for Experimental Medicine, Queen’s University Belfast, 97 Lisburn Road, Belfast, BT9 7AE UK

**Keywords:** DNA methylation, RNA methylation, Epigenetic drugs, Diabetes, Coronary microvascular dysfunction, Heart failure

## Abstract

Coronary microvascular dysfunction (CMD) is an established pathological driver of heart failure, with endothelial cell (EC) dysfunction representing a central determinant of its development and progression. EC impairment disrupts normal coronary microvascular tone and perfusion, and promotes myocardial inflammation, fibrosis, and cardiomyocyte stress, as characteristic features of the diabetic heart. CMD is often clinically silent and undiagnosed, whilst conventional therapies targeting cardiometabolic risk factors are largely ineffective towards restoring microvascular integrity. Emerging evidence implicates epigenetic dysregulation, including DNA and RNA methylation, as a critical mechanism underlying maladaptive EC signalling. These key modifications encode microvascular memory, sustaining endothelial dysfunction even after risk factors are optimally controlled; DNA methylation stabilises pathogenic transcription whilst RNA methylation regulates transcript stability and translation to support continued disruption of EC homeostasis. Indeed, preclinical studies demonstrate that pharmacological DNA methylation inhibitors and RNA methylation modulators can restore healthy EC function, reflected by reduced inflammation and preserved microvascular integrity, positioning such epigenetic pathways as central determinants of CMD with clear mechanistic relevance and therapeutic potential. Selective methylation targeting therefore offers exciting translational promise to reprogram dysfunctional ECs in diabetic patients, reversing CMD and preventing development and progression of associated heart failure. This timely review summarises current knowledge of EC epigenetic regulation, focusing on methylation modifications, and explores how emerging mechanistic insights may be leveraged to advance therapeutic targeting of CMD in the diabetic heart as foundation for development of innovative clinical management strategies.

## Endothelial Dysfunction in the Diabetic Heart

Diabetes mellitus (DM) is a growing global health burden, currently impacting the lives of around 589 million adults worldwide and projected to affect 853 million people by 2050 [1]. Cardiovascular disease (CVD) is a major complication of diabetes and leading cause of morbidity and mortality, due to diabetic cardiomyopathy, myocardial infarction, stroke, and heart failure [2, 3]. Importantly, diabetes-related cardiometabolic stress contributes to widespread microvascular dysfunction, including within the coronary circulation, leading to impaired myocardial perfusion through altered vasomotor regulation and vascular remodelling [4, 5]. Central to these processes are endothelial cells (ECs) lining the luminal surface of all blood vessels, are essential for maintaining vascular health whilst comprising approximately one-third of all cells in the human heart [6]. Through tightly regulated signalling pathways, ECs control key homeostatic functions, including vascular tone, blood flow, permeability, inflammation, coagulation, and angiogenesis [7]. Endothelial dysfunction, primarily driven by reduced nitric oxide (NO) bioavailability and hyperpolarisation, impairs these critical processes and is exacerbated by established cardiovascular risk factors, including diabetes, hyperlipidaemia, aging, obesity, hypertension, smoking, chronic inflammation, and genetic susceptibility [8, 9]. Compromised endothelial regulation promotes inadequate microvascular perfusion, resulting in impaired tissue oxygen and nutrient delivery as well as inefficient waste removal, impacts which are especially detrimental to the heart as a highly metabolic organ with low tolerance to ischaemia [10, 11].

**Fig. 1:**
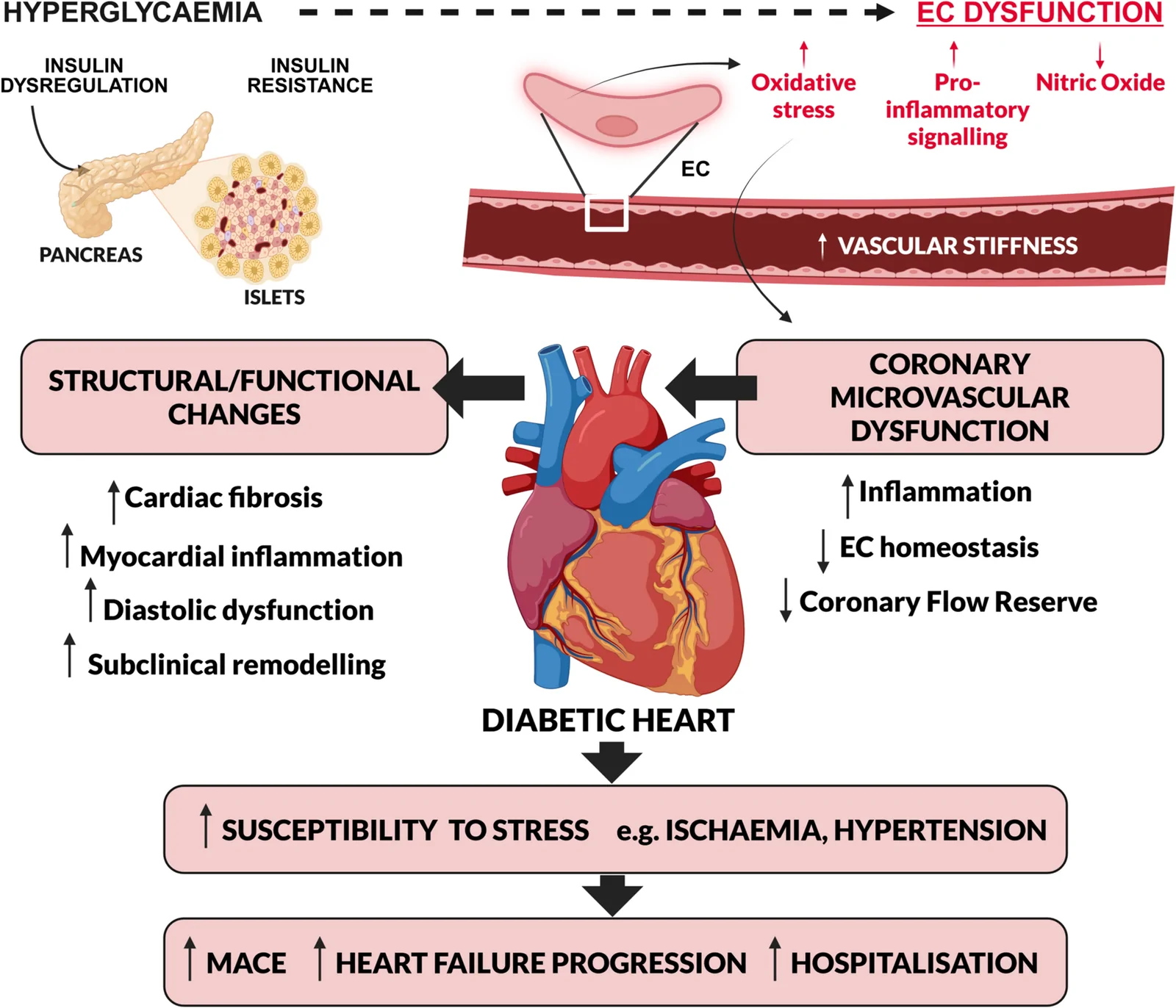
Hyperglycaemia-driven coronary microvascular dysfunction and heart failure in diabetes. Chronic hyperglycaemia (due to insulin dysregulation and resistance) promotes endothelial cell (EC) oxidative stress, pro-inflammatory signalling, and endothelial dysfunction. These pathological stimuli impair endothelial NO bioavailability, leading to reduced endothelium-dependent vasodilation and increased vascular stiffness. Coronary microvascular ECs are particularly vulnerable to hyperglycaemic stress, resulting in coronary microvascular dysfunction (CMD) and impaired myocardial perfusion which drives diastolic dysfunction and adverse cardiac remodelling particularly characterised by myocardial fibrosis, inflammation, and extracellular matrix deposition. Collectively, these changes predispose to chronic cardiovascular stresses, such as ischaemia and hypertension, increasing risk of MACE and exacerbating heart failure progression and hospitalisation

In addition to its systemic metabolic effects, diabetes exerts profound structural and functional alterations on the coronary microcirculation that contribute to initiation and progression of diabetic heart disease [12, 13]. Hyperglycaemia, oxidative stress and inflammation all decrease nitric oxide bioavailability and endothelium-dependent vasodilation, leading to increased vascular stiffness, impaired coronary flow reserve, and coronary microvascular dysfunction (CMD). The diabetic heart is particularly characterised by capillary rarefaction, basement membrane thickening, disruption of the endothelial glycocalyx, increased permeability, and perivascular fibrosis. Together, these changes lead to decreased myocardial oxygen supply, inflammatory cell infiltration, extracellular matrix deposition, cardiomyocyte remodelling, and diastolic dysfunction [14]. Microvascular dysfunction further contributes to disease progression via activation of key pathological drivers, such as extravasation of circulating inflammatory cells and endothelial-to-mesenchymal transition (EndMT), as characteristic features of the diabetic heart [15]. Collectively, these microvascular abnormalities establish a pathogenic substrate that predisposes diabetic patients to major adverse cardiovascular events (MACE), including myocardial infarction and heart failure, with CMD emerging as a key determinant of adverse prognosis [16, 17]. Development and progression of CMD and heart failure in diabetes is summarised in Fig. 1.

## Coronary Microvascular Dysfunction: Gaps in Detection and Clinical Management

Despite its clinical significance, CMD is often undiagnosed and untreated, with no approved evidence-based therapies directly targeting the coronary microvasculature [12]. CMD may be clinically silent with some individuals presenting with non-specific symptoms such as chest pain, fatigue, and shortness of breath [18]. Standard diagnostic approaches in such patients, including coronary angiography, often appear normal and fail to detect microvascular issues, with accurate diagnosis typically requiring specialised assessments, such as coronary flow reserve and cardiac magnetic resonance imaging, which are often inaccessible [18, 19]. This clinical challenge is further exacerbated by lack of validated biomarkers which are specific for CMD, thereby limiting opportunity for reliable non-invasive detection together with accurate monitoring of disease progression and therapeutic response [20]. Moreover, first line cardiovascular therapies, such as angiotensin-converting enzyme inhibitors and β-blockers, offer limited ability to impact coronary microvascular function. Although emerging pharmacological strategies, such as sodium–glucose cotransporter 2 inhibitors and glucagon-like peptide-1 receptor agonists, which are now widely prescribed in diabetes, together with structured lifestyle interventions, show promise with regard to CMD, therapeutic efficacy and functional responses remain sub-optimal [21–23]. This treatment gap underscores urgent need for precision, EC-targeted diagnostics and therapeutics to directly target key molecular pathological drivers of CMD, moving beyond symptom relief towards management strategies that can positively impact microvascular function.

### Diabetes-Linked Epigenetic Endothelial Dysregulation: Implications for Coronary Microvascular Dysfunction

In diabetes, CMD significantly contributes to the development and progression of associated cardiovascular complications [10] with a significant proportion of individuals developing characteristic cardiomyopathy [1, 2]. Emerging evidence indicates that epigenetic modifications, which regulate gene expression without altering DNA sequence, are key determinants of EC function [24, 25], representing a potential mechanism underlying chronic CMD in diabetes which often persists in optimally-treated patients. This is linked to the concept of EC metabolic memory, whereby early exposure to metabolic stresses, such as hyperglycaemia, triggers molecular alterations that persist even after normalisation of blood glucose levels, locking ECs in a maladaptive state which promotes sustained vascular dysfunction [26–28]. Among established epigenetic mechanisms, DNA and RNA methylation represent particularly compelling modifications for potential therapeutic intervention, given their reversible nature [29, 30]. Although epigenetic therapies have been primarily developed for oncology, several of which have received FDA approval [31], accumulating evidence highlights their emerging potential for cardiometabolic disease. Intriguingly, mechanistic insights gained from non-cardiac ECs can provide important information to promote detailed understanding of epigenetic signalling in coronary microvascular ECs, towards development of targeted therapies and clinical management strategies to alleviate CMD in diabetic patients. It is important to acknowledge that histone modifications represent a significant additional layer of epigenetic regulation, with multiple histone methyltransferases and demethylases implicated in CVD progression [32]. Of particular note, in the context of the current article, histone acetylation, a key regulatory modification, is reported to interact with DNA methylation and influence gene expression through effects on chromatin architecture [33]. However, we chose to focus our rev primarily on DNA and RNA methylation, owing to their relative stability and emerging relevance as therapeutic targets in CVD, with histone modifications well reviewed elsewhere [32, 34].

### DNA Methylation in Endothelial Cells

DNA methylation is the most biologically stable and extensively characterised epigenetic modification, occurring at CpG (Cytosine-phosphate-Guanine) sites across the mammalian genome [24, 35]. Within promoter regions, DNA methylation in suppresses gene expression by preventing transcription factor binding to DNA. This process is mediated by a family of enzymes known as DNA methyltransferases (DNMTs), among which DNMT1 functions primarily in maintenance methylation, while DNMT3A and DNMT3B catalyse *de novo* methylation, particularly during embryonic development [24]. Other renowned enzymes involved in DNA methylation regulation are TET (Ten-eleven translocation) dioxygenases, which reverse DNA methylation by oxidizing the methyl group into several derivatives [36, 37]. It is important to note that activity of these enzymes is influenced by the cellular environment, leading to higher activity under certain pathophysiological insults. Specifically, aberrant laminar shear stress due to disturbed blood flow increases DNMT1 expression, linking pathologies such as atherosclerosis to hypermethylation [38]. With particular relevance to diabetes, increased DNMT1 activity is reported to drive metabolic stress in endothelial cells under disturbed-flow [39], whilst low shear stress downregulates TET2 under atherosclerotic conditions, further promoting a hypermethylated state via reduced activity of this DNA methylation ‘eraser’ which also drives EndMT-mediated endothelial dysfunction [40].

Multiple studies have demonstrated that hyperglycaemia can epigenetically reprogramme ECs, mechanistically linking metabolic stress to endothelial dysfunction and vascular pathology [25]. Exemplifying this, Pepin and colleagues reported that exposure of human aortic ECs to hyperglycaemia induced methylation in > 2000 genes linked to key endothelial processes such as angiogenesis and NO signalling [41]. Notably, hyperglycaemia-induced DNA methylation changes appear to be largely coordinated by DNMT1, positioning this enzyme as a central mediator of glucose-driven endothelial reprogramming and focal point for understanding and targeting vascular dysfunction. For example, hyperglycaemic culture of retinal ECs induced DNMT1 expression and activity, which was reversed by pharmacological inhibition with 5-aza-2′-deoxycytidine (5-aza-dC), in parallel with restored expression of key antioxidants, MnSOD and GSTT1, and suppression of inflammation (e.g. intercellular adhesion molecule 1; ICAM-1). Importantly, these in vitro findings were validated in retinal tissue isolated from diabetic rats as evidence of pathological relevance [42]. Similarly, high glucose culture of human umbilical vein ECs (HUVECs) induced DNMT1 expression and DNA methylation-dependent reprogramming of downstream gene transcription, together with 5-aza-dC-sensitive enrichment of secreted exosomes with key vascular injury-associated proteins, including thrombospondin 1 (TSP-1), pentraxin 3 (PTX3), and cystatin C (CysC) [43]. Such findings support DNMT1-mediated DNA methylation as a central driver of maladaptive EC signalling in diabetes, promoting a pro-inflammatory and pro-oxidative environment whilst also disrupting normal paracrine communication. Consistent with this hypothesis, hyperglycaemia is reported to negatively impact genomic imprinting in retinal ECs by promoting aberrant DNA methylation, mediated by DNMT1 and the imprinted long non-coding RNA, MEG3, which maintains endothelial homeostasis [44, 45]. Genomic imprinting is a critical epigenetic process within which only the maternal or paternal allele is expressed, ensuring effective regulation of genes involved in key EC pathways linked to metabolism, cell growth and vascular function [44–47]. Indeed, hyperglycaemia-induced repression of MEG3 was found to activate PI3K/Akt/mTOR signalling, triggering EndMT as a central transducer of downstream cardiac remodelling, whilst overexpression of MEG3 suppressed EndMT and restored normal EC function [45]. Taken together, these findings highlight an important role for DNMT1/DNA methylation in regulation of protective imprinted genes, whilst suggesting that loss of imprinting via DNMT1-mediated methylome remodelling may play a pivotal role in determining CMD.

Notably, stability of DNA methylation confers its capacity to encode long-term pathogenic memory, which may be largely responsible for sustaining aberrant endothelial gene expression even after normalisation of glycaemia, positioning this key epigenetic modification as a likely central driver of persistent CMD [26, 27]. Indeed, DNA methylation has been proposed to underlie metabolic memory, whereby previous exposure to hyperglycaemia may lead to chronic endothelial damage [48]. Specifically, DNA methylation signatures at individual CpG sites have been linked to previous glycaemic exposure and correlated with development of diabetic complications [49], strongly suggesting that persistent DNA methylation alterations in ECs may serve as a molecular marker of long-term cardiovascular risk. In this regard, human aortic ECs exposed to high glucose show gene-specific DNA methylation changes which are evident for at least 48 h after return to normal glucose conditions, with hyperglycaemia-induced hypomethylation of the SIRT6 promoter reflected by elevated SIRT6 expression [50]. This may be significant in the context of CMD associated with diabetes as SIRT6 is known to support vascular homeostasis, so DNA methylation-mediated upregulation could represent a cardioprotective response [51]. Regardless of directional impact on gene expression, persistence of promoter DNA methylation and elevated DNMT1 activity further highlights importance of this epigenetic modification in sustaining long-term EC responses to glycaemic stress and determining (mal)adaptive methylome remodelling and detrimental cardiovascular impacts.

Intrauterine and early post-natal exposure to elevated glucose levels are crucial factors that can reprogramme endothelial epigenetic status, predisposing to persistent vascular dysfunction and elevated cardiovascular risk. This is evidenced by reports that dysfunctional HUVECs from donors with gestational diabetes or type 2 diabetes show increased global DNA methylation and DNMT3B expression versus those isolated from healthy controls [52]. Furthermore, integrated methylome and transcriptome analysis in dysfunctional fetoplacental arterial ECs exposed to gestational diabetes identified differential methylation and increased expression of ABR (active BCR-related gene), a RhoA activator involved in actin cytoskeleton organisation, silencing of which restored normal RhoA activity and endothelial capacity [53]. Such observations demonstrate that DNA methylation changes which are established *in utero* may persist as likely foundation for later susceptibility to EC dysfunction, including CMD associated with diabetes. A striking example of chronic impact of *in utero* stress linked with aberrant DNA methylation is provided by children from the Dutch Hunger Winter cohort who were exposed to famine during embryonic development. These individuals displayed persistent locus-specific DNA methylation of several genes with metabolic relevance, including imprinted insulin-like growth factor 2 (IGF2), which was detectable in adulthood and linked with chronic dyslipidaemia, impaired glucose homeostasis, and accelerated biological aging [54, 55]. A further cohort study spanning over 40 years has linked maternal diabetes to increased CVD risk in offspring during early life stages. It is likely that this phenomenon is at least partly due to glucose-induced epigenetic reprogramming during fetal development, pointing to existence of long-term epigenetic memory that may not only influence health at birth but also manifest later in life [56, 57].

Whilst mounting evidence underscores importance of DNMT1-mediated DNA methylation in ensuring appropriate expression of protective endothelial genes, effective balance between DNA methylation and demethylation is essential to maintain vascular homeostasis [58]. TET enzymes, which are central to demethylation, are pivotal in actively removing DNA methylation, thereby preventing maladaptive DNA alterations and consequent endothelial dysfunction [59]. Supporting this concept, emerging evidence reveals that dysregulation of TET activity is linked to diabetes-induced endothelial dysfunction. For example, RNA sequencing of lung ECs from TET2 knockout mice revealed marked dysregulation of inflammatory response linked with interferon signalling, with 70% of transcriptionally impacted genes also altered in endothelium of diabetic mice [60]. Pathway analysis of diabetic EC mRNA expression also highlighted interferon signalling as highly dysregulated versus non-diabetic ECs, further supporting likely importance of TET2 disruption in driving altered EC signalling in diabetes [60]. Similarly, TET2 is reported to regulate vascular-specific genes, such as ROBO4, by mediating active demethylation of its promoter, linked with accelerated development of diabetic retinopathy [61], whilst TET2 deficiency impairs HUVEC angiogenesis via suppression of STAT3 signalling [62]. Collectively, these findings highlight TET enzymes, particularly TET2, as central regulators of endothelial epigenetic balance, linking DNA methylation dynamics to metabolic stress and vascular dysfunction in diabetes. As such, they may hold potential for therapeutic development through targeting of key methylation pathways, including ROBO4 and STAT3.

Taken together, current literature adds significant support to the hypothesis that persistent DNA methylation alterations in ECs encode metabolic memory as a likely critical contributor to CMD development and progression. Importantly, emerging evidence indicating that DNMT1-mediated DNA methylation may sustain vascular dysfunction in diabetes even with optimised glycaemic control positions these epigenetic marks both as potential innovative therapeutic targets for CMD in diabetes and biomarkers to facilitate early disease detection, risk stratification, and treatment monitoring.

### Targeting DNA Methylation: A Promising Strategy Against Coronary Microvascular Dysfunction

Pharmacological targeting of DNA methylation pathways using DNMT1 inhibitors, such as 5-aza-dC, offers a promising translational strategy to restore endothelial function and reduce progression of diabetic microvascular complications such as CMD. Although specific epigenetic mechanisms driving EC dysfunction in diabetes remain incompletely defined and DNMT1 inhibitors are yet to be explored in directly relevant experimental models, extensive preclinical studies have demonstrated that DNA methylation modulation can enhance vascular health. Such findings provide strong rationale for investigating DNMT1-targeting strategies to alleviate CMD, with 5-aza-dC (decitabine), an FDA-approved treatment for myelodysplastic syndromes and acute myeloid leukaemia, amongst the most widely studied candidates [63]. With direct relevance to cardiovascular disease and CMD, low-dose 5-aza-dC reduces atherosclerotic lesions in Ldlr⁻/⁻ mice, in parallel with suppressed inflammation reflected by decreased macrophage infiltration and expression of proinflammatory cytokines (e.g. TNF-α, IL-6). Mechanistically, 5-aza-dC exposure promotes demethylation of liver X receptor α (LXRα) and peroxisome proliferator-activated receptor γ1 (PPARγ1), driving increased gene expression and anti-inflammatory and atheroprotective impacts relevant to EC signalling [64]. Inhibition of DNA methylation is also reported to reverse cardiac hypertrophy and dysfunction in vivo. For example, 5-aza-dC post-treatment in a rat model of norepinephrine–induced hypertension reduces global hypermethylation and altered left ventricular protein expression, whilst alleviating cardiac hypertrophy, contractile dysfunction, and susceptibility to ischaemic damage, indicating that increased DNA methylation directly drives adverse cardiac remodelling [65]. Further experimental studies highlight exciting therapeutic potential of demethylating agents, such as 5-aza-dC, for specific targeting of immune-mediated and endothelial signalling as central determinants of CMD. For example, 5-aza-dC was shown to induce differentiation of naïve CD4⁺ T cells to regulatory T cells (Tregs), as critical suppressors of immune activation and inflammation, whilst adoptive transfer of Tregs into ApoE⁻/⁻ mice reduced systemic inflammation and atherosclerosis development. Mechanistically, 5-aza promotes DNMT1-dependent loss of DNA methylation at the Foxp3 Treg-specific demethylated region (TSDR), resulting in upregulation of Foxp3 expression and accelerated conversion of naive CD4 + T cells into functional Tregs. Taken together, these findings clearly demonstrate that DNMT1-targeted demethylation can modulate immune capacity and confer EC protection against disease progression [66]. Complementary research indicating that 5-aza-dC treatment normalises elevated plasma homocysteine and blood pressure in hyperhomocysteinaemic rats is particularly intriguing as homocysteine is known to influence DNA methylation [67, 68], suggesting that vascular benefits of DNA methylation inhibition may at least partly result from direct effects on homocysteine-mediated methylome remodelling. Further support for therapeutic potential of cardiovascular DNA methylation targeting is provided by studies which applied low-dose 5-aza treatment in spontaneously hypertensive rats and mice subjected to chronic pressure-overload to reduce elevated global DNA methylation and DNMT1 expression, resulting in attenuated cardiac hypertrophy, fibrosis, and diastolic dysfunction [69, 70]. These observations are compelling towards highlighting specific targeting of DNA methylation pathways as a viable strategy to prevent or reverse adverse cardiac remodelling, with direct relevance to CMD where epigenetic alterations are likely to contribute to EC dysfunction and disease progression. Indeed, pretreatment of bone marrow progenitor cells with 5-aza-dC is reported to promote enhanced EC differentiation in vitro, whilst of 5-aza-dC-primed cells injection into infarcted mouse improved functional recovery versus naïve cells [71].

Whilst 5-aza-dC incorporates into DNA during replication, covalently trapping DNMTs to induce passive DNA demethylation, other DNMT1-targeting drugs act via alternate mechanisms [72]. For instance, RG108 [2-(1,3-dioxoisoindolin-2-yl)−3-(1 H-indol-3-yl) propanoic acid], a non-nucleoside DNMT1 inhibitor, binds directly to the catalytic site of DNMT1 without incorporating into DNA, thereby inhibiting enzymatic activity and avoiding cytotoxicity associated with 5-aza-dC [73]. Despite this different mechanism of action, RG108 also shows therapeutic promise for cardiovascular disease, demonstrated by reduction of cardiac hypertrophy and fibrosis in a rat pressure-overload model and prevention of stress-induced contractile dysfunction, effects mediated at least partly through demethylation [74]. It is important to note that whilst RG108 offers enhanced selectivity and reduced cytotoxicity, covalent trapping of DNMTs by 5-aza-dC promotes long-lasting impacts on epigenetic signalling, making it better suited to reset maladaptive DNA methylation linked with chronic cardiometabolic disease and provide sustained therapeutic benefit. In this regard, selective DNA methylation targeting offers clear potential to restore or protect EC homeostasis, thereby preventing development and progression of CMD in diabetes. Table 1 summarises key reported functional impacts of DNA methylation targeting with DNMT1 inhibitors, 5-aza-dC and RG108, as important preclinical evidence to support clear translational potential of these drugs for clinical management of CMD.

**Table 1:** Summary of reported functional impacts of methylation targeting in preclinical disease models with direct relevance to coronary microvascular dysfunction

DNA METHYLATION				
Study	Drug/Target	Disease Focus	Preclinical Model	Functional Impact
Cao et al. [64]	5-aza-dC	Atherosclerosis	Ldlr⁻/⁻ mice	↓ lesion development ↓ macrophage infiltration ↓ inflammatory signalling
Xiao et al. [65]	5-aza-dC	Cardiac hypertrophy	Norepinephrine-induced hypertension (rat)	↓ cardiac hypertrophy ↓ contractile dysfunction ↓ susceptibility to ischaemia
Zhu et al. [66]	5-aza	Atherosclerosis	ApoE⁻/⁻ mice	↑ CD4 + differentiation ↓ inflammation ↓ lesion development
Narayunan et al. [67]	5-aza-dC	Hyperhomocysteinaemia	CBS^+/−^ mice	↓ plasma homocysteine ↓ hypertension
Watson et al. [69]	5-aza	Heart failure	Spontaneously hypertensive rat	↓ cardiac hypertrophy ↓ cardiac fibrosis ↓ diastolic dysfunction
Russell-Hallinan et al. [70]	5-aza	Heart failure	Transverse aortic constriction (mouse)	↓ cardiac hypertrophy ↓ cardiac fibrosis ↓ diastolic dysfunction
Rajasingh et al. [71]	5-aza-dC	Myocardial infarction	Coronary artery ligation (mouse)	↑ bone marrow progenitor cell-induced functional recovery
Stenzig et al. [74]	RG108	Heart failure	Transverse aortic constriction (rat)	↓ cardiac hypertrophy ↓ cardiac fibrosis ↓ contractile dysfunction
**RNA METHYLATION**				
**Study**	**Drug/Target**	**Disease Focus**	**Preclinical Model**	**Functional Impact**
He et al. [96]	METTL3	Myocardial infarction	Coronary artery ligation (mouse)	↓ cardiac fibrosis ↓ cardiac dysfunction
Cao et al. [97]	METTL14	Heart failure	Doxorubicin-induced cardiotoxicity (mouse)	↓ myocardial injury ↓ cardiac dysfunction ↓ adverse remodelling
Huang et al. [100]	FTO	Cancer	AML cell xeno-transplantation (mouse)	↓ cancer cell proliferation

In addition to therapeutic inhibition of DNMT1, intriguing evidence is emerging to suggest that TET enzymes, which regulate DNA demethylation, are highly relevant to endothelial homeostasis and vascular dysfunction [60–62]. Whilst TET-targeted therapies have not yet advanced to clinical development, specific modulation of TET activity or precision epigenome editing to promote demethylation represent promising translational strategies to restore DNA methylation balance and endothelial homeostasis. In this regard, insights from the oncology field provide compelling support for TET-based therapeutic targeting [75]. For example, robust leukaemia cell line screening identified mitoxantrone as a potent TET agonist which promoted TET-dependent cell death, highlighting TET-mediated demethylation as a critical determinant of reported anti-cancer effects [76]. Although TET-targeting drugs remain limited to preclinical investigation, such findings indicate that modulation of TET activity can alter epigenetic states linked to cell survival, thereby holding translational potential for clinical oncology. Importantly, this principle also provides mechanistic rationale for exploring TET modulation in the context of endothelial homeostasis and vascular disease, particularly in settings such as diabetes where DNA methylation balance is disrupted. By restoring dynamic interplay between methylation and demethylation, TET-targeted strategies may complement more established DNMT1 inhibition to confer enhanced protection of endothelial function and reduced CMD progression.

### RNA Methylation in Endothelial Cells

Alongside DNA methylation, RNA methylation represents a particularly important epigenetic modification in the context of CMD. RNA methylation dynamically modulates gene expression in response to metabolic and environmental stress, and has been indicated to influence pathways contributing to CMD development and progression [30, 77, 78]. Specifically, RNA methylation involves addition of a methyl group to RNA molecules, including messenger RNA (mRNA), the most common form being N6-methyladenosine (m⁶A). In a manner analogous to DNMTs in DNA methylation, METTL3 and METTL14 function as the primary RNA methyltransferases (‘writers’) responsible for catalysing m⁶A modification, whilst fat mass and obesity-associated protein (FTO) serves as an ‘eraser,’ promoting their removal and thereby contributing to maintenance of RNA stability and function [79–81].

RNA methylation serves to control RNA stability, splicing, translation efficiency, and decay, supporting fine-tuning of gene expression and providing an effective mechanism by which cells rapidly respond to environmental or metabolic signals [77, 82, 83]. Importantly, emerging evidence implicates dysregulation of RNA methylation in endothelial dysfunction, highlighting a likely critical role in CMD, with expression of key RNA methylation regulators linked to disruption of EC gene expression and signalling in diabetes [78, 84, 85]. For example, human retinal vascular ECs cultured under hyperglycaemic conditions show decreased expression of METTL3, which mediates m⁶A modification of RNA transcripts [86], whilst HUVECs exposed to high glucose show reduced METTL3 expression and m⁶A RNA methylation linked to aberrant Cds2-mediated metabolism and angiogenic response [87]. These studies indicate a likely important role for RNA methylation in driving EC dysfunction in diabetes, which can be reversed upon restoration of METTL3, providing further support for this hypothesis.

With particular relevance to CMD in diabetes, loss of METTL3-mediated m⁶A formation is reported to disrupt proliferation, migration and EndMT in high glucose-treated HUVECs, as critical determinants of characteristic cardiac remodelling [88], highlighting an apparent specific role for RNA methylation in this setting. Similarly, m⁶A methylation of long non-coding RNAs, which is important for maintaining their stability, localisation, and translation, is negatively impacted in HUVECs exposed to hyperglycaemia and linked with disruption of EC gene expression and function [89]. Indeed, in human retinal microvascular ECs cultured under diabetic conditions, FTO expression is upregulated whilst global m⁶A levels are oppositely decreased, driving EC inflammation. Similarly, mice with endothelial-specific knockout of FTO subjected to experimental diabetes showed reduced retinal vascular leakage and acellular capillary formation, driven by NF-κB–mediated inflammation due to FTO-mediated removal of m⁶A marks from TNIP1 mRNA and consequently reduced expression [90]. Complementary analysis of high glucose-treated HUVECs demonstrated that FTO induction promotes aberrant m⁶A modification and expression of HOXA9 and PLAU as key EC regulators, providing additional mechanistic evidence that RNA methylation contributes to endothelial dysfunction in diabetes [91]. Whilst m⁶A ‘writers’ and ‘erasers’ are central to establishment and removal of RNA methylation marks, m⁶A ‘readers’ appear to be equally important in diabetes [92]. For example, the m⁶A reader, heterogeneous nuclear ribonucleoprotein C (HNRNPC), is upregulated in HUVECs under diabetic conditions and linked with disruption of vascular homeostasis through specific activation of PSEN1-Notch signalling and reduced NO generation [93]. Furthermore, increased expression of the m⁶A reader, YTH domain-containing protein 1 (YTHDC1), in human retinal vascular ECs exposed to hyperglycaemia, impairs cell viability, proliferation, migration, and tube formation, which is rescued by restoration of CDK6 expression, highlighting the YTHDC1-CDK6 axis as a critical regulator of EC endothelial homeostasis and potential therapeutic target in diabetes [94]. Collectively, these observations provide compelling evidence that dysregulated RNA methylation, mediated by a combination of m⁶A writers, erasers, and readers, directly drives endothelial dysfunction in diabetes. As such, it appears that epitranscriptomic alterations in diabetic ECs disrupt key homeostatic signalling processes [95], thereby promoting functional abnormalities that are likely to significantly contribute to CMD and associated adverse cardiac remodelling.

### Targeting RNA Methylation: A Promising Strategy Against Coronary Microvascular Dysfunction

RNA methylation, particularly m⁶A, has emerged as a central driver of endothelial dysfunction in diabetes, with direct translational relevance to CMD. Whilst therapeutic targeting of DNA methylation has been advanced to clinical treatment of myelodysplastic syndromes and acute myeloid leukaemia, human trials for RNA methylation targeting have yet to be conducted. Nonetheless, a growing body of preclinical data supports translational potential of RNA methylation-based cardiovascular strategies [30]. For example, METTL3-mediated m⁶A levels are elevated in mouse hearts subjected to chronic coronary artery ligation and in isolated mouse cardiac fibroblasts exposed to hypoxia, and contribute to myocardial collagen deposition, cardiac dysfunction, and fibroblast proliferation/differentiation via stabilisation of SMOC2 mRNA stabilisation [96]. Similarly, METTL14 is upregulated in doxorubicin-induced heart failure, resulting in aberrant m⁶A modification of various mRNAs, specific induction of miR-221-3p, and cardiomyocyte cell death [97], providing additional support for RNA methylation as a potential therapeutic target for adverse cardiac remodelling. Importantly, expression of RNA methylation modifiers, METTL14 and FTO, increases with age as a critical determinant of cardiac dysfunction, due to aberrant m⁶A at genes regulating central pathological pathways such as PI3K-Akt signalling, with specific m⁶A-driven enhancement of EFEMP1 (linked to onset of senescence) [98]. It is interesting to note apparent induction of FTO in aged hearts as a likely compensatory response against maladaptive m⁶A accumulation. Consistent with this observation, cardiac expression of the demethylase, FTO, is decreased in a mouse model of acute LPS-induced endotoxaemia characterised by contractile dysfunction, whilst m⁶A-mediated RNA methylation is increased together with elevated proinflammatory cytokines (IL-6, TNF-α, IL-1β). These effects were reproduced by FTO knockdown in rat H9c2 cardiomyoblasts [99]. As was initially the case for DNA methylation, therapeutic potential for pharmacological targeting of m⁶A RNA methylation is strongly supported by development of small-molecule modulators in the oncology field. In this regard, selective inhibitors of both FTO (e.g. meclofenamic acid, FB23-2, Dac51) and METTL3 (STM2457) have demonstrated robust efficacy to promote m⁶A modulation in multiple preclinical cancer models, positively impacting cell proliferation, differentiation, and survival, with some of these compounds advancing to early-phase clinical trials [100]. Such translational success provides compelling proof-of-principle evidence that RNA methylation is amenable to pharmacological manipulation which can reduce disease progression. Although these drugs are yet to be evaluated in a clinical cardiovascular context, their ability to precisely modulate m⁶A both in vitro and in vivo, together with the reported cardiovascular benefits of 5-aza-dC, highlights selective targeting of RNA methylation as a potentially viable approach to promote EC health and reduce CMD and heart failure progression in diabetes. Table 1 summarises key reported functional impacts of RNA methylation targeting using both METTL and FTO inhibitors, as emerging preclinical evidence supporting potential cardiovascular utility of such drugs.

## DNA and RNA Methylation as Key Drivers of Coronary Microvascular Dysfunction: Translational Perspectives

### Evidence from Cardiovascular Research and Challenges with Epigenetic Targeting

CMD is a central driver of impaired myocardial perfusion, ischaemia, and heart failure, and a particularly important determinant of disease progression in diabetes [12]. Accumulating evidence highlights epigenetic mechanisms, specifically DNA methylation and RNA methylation, as key regulators of microvascular health [64, 88]. Whilst DNMT1-maintained DNA methylation possesses capacity to encode durable ‘metabolic memory,’ resulting in sustained suppression of protective genes [27], RNA methylation, particularly m⁶A, confers dynamic post-transcriptional regulation, allowing fine-tuning of endothelial responses to both metabolic and inflammatory stress [78]. Together, it is evident that these complementary mechanisms play an important role in maintaining both EC homeostasis and maladaptive signalling with capacity to sustain development and progression of CMD, even with optimal management of established risk factors such as hyperglycaemia. Indeed, pharmacological targeting of DNA methylation with specific DNMT1 inhibitors, such as 5-aza-dC or 5-azacytidine, which are used clinically for cancer treatment [101], appears to represent a promising strategy to protect EC and microvascular homeostasis and prevent CMD. In this regard, preclinical studies have clearly demonstrated that DNA methylation modulation enhances vascular health by suppressing maladaptive signalling, thereby mitigating adverse cardiac remodelling. For example, low-dose 5-aza-dC reduces inflammation and atherosclerotic lesion development, together with pressure-overload and hypertensive cardiac remodelling in various animal models by specific reversing disease-induced hypermethylation [64–67, 70, 71]. Beyond conventional small molecule inhibitors (e.g. 5-aza-dC), selective DNMT1 inhibitors (e.g. RG108) offer significant potential for precise modulation of epigenetic signalling, thereby minimising off-target effects [73]. By resetting diabetes-associated maladaptive DNA methylation patterns in coronary microvascular ECs, such strategies could directly address specific mechanisms underlying CMD and heart failure (rather than managing symptoms with standard cardiovascular drugs) by alleviating persistent proinflammatory and prooxidative signalling to protect and ultimately restore EC homeostatic function.

Despite clear promise of epigenetic therapies in both experimental and clinical settings, translation of DNA and RNA methylation-targeted epigenetic drugs for CMD remains largely unexplored. Although DNA methylation inhibitors are FDA-approved for treatment of myelodysplastic syndromes and acute myeloid leukaemia, their use may be associated with systemic toxicity due to off-target oncogene activation and genome destabilisation secondary to global hypomethylation [101–103]. Long-term safety of non-specific epigenetic targeting is a serious concern as sustained DNA manipulation may significantly increase risk of cancer and other unpredictable systemic impacts, which is particularly relevant to potential cardiovascular applications. With regard to CMD, these challenges are amplified by the unique pathobiology of coronary microvascular ECs, which have distinct metabolic demands and signalling compared to other EC populations. As mechanistic insights into EC epigenetic regulation are largely derived from the latter, including aortic, umbilical vein, retinal, and fetoplacental ECs, it is not clear whether currently identified DNA and RNA methylation targets may be directly relevant to CMD, raising concerns of potential off-target effects on other cell types driving unintended epigenetic alterations and systemic toxicity. Delivery of epigenetic therapies for CMD may also be challenging as it is important that drugs are targeted to coronary microvascular ECs at effective concentrations without negatively impacting other vascular beds and tissues. In this regard, innovative delivery methods, such as nanoparticles, targeted liposomes, or viral vectors, are currently under development to improve drug targeting and efficacy [104–106], although these approaches remain largely untested in clinical practice due to persistent safety concerns. Adding a further layer of complexity, the interconnected nature of epigenetic networks presents another challenge to targeted intervention, due to likely compensatory signalling that may reduce drug efficacy. It is therefore important that future studies adopt a multi-omics approach to examine combined impacts of DNA and RNA methylation and transcriptional regulation on CMD in diabetes to identify underlying mechanisms with direct therapeutic relevance [107, 108].

### Limitations of Current Studies and Challenges in Translating Findings to CMD

It is important to consider that most of the available evidence for epigenetic modification as an important determinant of EC signalling in diabetes comes from non-cardiac cells or cardiac disease models which are not directly relevant to clinical CMD. Relative paucity of studies using human coronary microvascular ECs or animal models specifically characterised by CMD creates some uncertainty about which epigenetic targets may be most appropriate for translation, thereby limiting identification of therapeutic candidates. It is therefore critical to conduct appropriately designed experiments using clinically relevant cell and animal models which faithfully recapitulate human coronary microvascular biology to gain more complete understanding of how DNA and RNA methylation determine CMD, incorporating CRISPR-based epigenetic editing to target specific methylation marks as foundation for therapeutic translation [109–112]. Such approaches are essential to uncover specific methylation patterns contributing to maladaptive gene expression and EC dysfunction in the context of CMD and diabetes towards development of targeted interventions circumventing toxicity associated with conventional pharmacological strategies. Whilst DNMT1 inhibitors, 5-aza and 5-aza-dC, have been shown to confer beneficial vascular effects in pre-clinical models, it is vital that future research directions include design of epigenetic therapies that preferentially impact gene-specific cell targets and minimise potential off target detrimental impacts.

### Emerging Approaches for Epigenetic Biomarker Discovery and Validation

Notably, there are currently no reliable methods for non-invasive assessment of CMD, typically requiring cardiac catheterisation for thermodilution analysis of microvascular resistance and coronary flow reserve, which is critical for both accurate diagnosis and monitoring of therapeutic responses [113]. In this regard, gaining understanding of how DNA and RNA methylation precisely impact CMD and drive adverse remodelling in the diabetic heart will not only shed light on key disease mechanisms but also offers intriguing possibility to support development of methylation-based biomarkers. Specifically, calculation of epigenetic risk scores (EpiScores), which combine DNA methylation changes across multiple genomic sites, hold potential to capture complex biological information reflecting key disease drivers, such as age and inflammation. With particular relevance to CMD and diabetes, EpiScores can be generated from blood DNA methylation patterns which act as proxies for circulating protein profiles and are capable of predicting cardiovascular, metabolic, and inflammatory outcomes [114]. Following this approach, there is clear scope for definition of CMD-specific methylation signatures, integrating key endothelial markers, angiogenic factors, and inflammatory mediators with functional validation, to support design of reliable diagnosis, risk stratification, and therapeutic monitoring. Such innovative methods could be complemented by Horvath’s epigenetic clock which combines information from multiple CpG sites to quantify biological age [115], with potential application to leverage methylation patterns as biomarkers of tissue and vascular aging which are closely linked with CMD. As such, epigenetic age acceleration offers a framework for understanding how cumulative methylation changes impact disease development and progression. Although relatively preliminary, these reports provide confidence that methylation-based biomarkers may realistically capture systemic drivers of CMD to support accurate risk stratification, highlighting evident importance of gaining detailed understanding of epigenetic dysregulation in this context. Whilst development of methylation-based biomarkers for CMD may be highly attractive, these are likely to face both biological and technical challenges. ECs are highly heterogenous, showing tissue specificity and altered methylation patterns in response to inflammation, low oxygen, blood flow, aging, and other disease stresses, making it difficult to distinguish population-specific changes [25, 116]. In addition, EC-derived DNA fragments are present at low levels in the blood and can resemble DNA from other cellular sources, making specific detection problematic [117, 118]. Both issues may be exacerbated by differences between individuals and limited availability of high-quality reference data for coronary microvascular ECs. Despite these challenges, accumulating biological evidence implicating DNA and RNA methylation as key disease drivers, together with rapid technical advancements, offer exciting and realistic potential for future design of methylation-based biomarkers to support effective clinical management of CMD and heart failure in diabetes.

### Future Perspectives

**Fig. 2:**
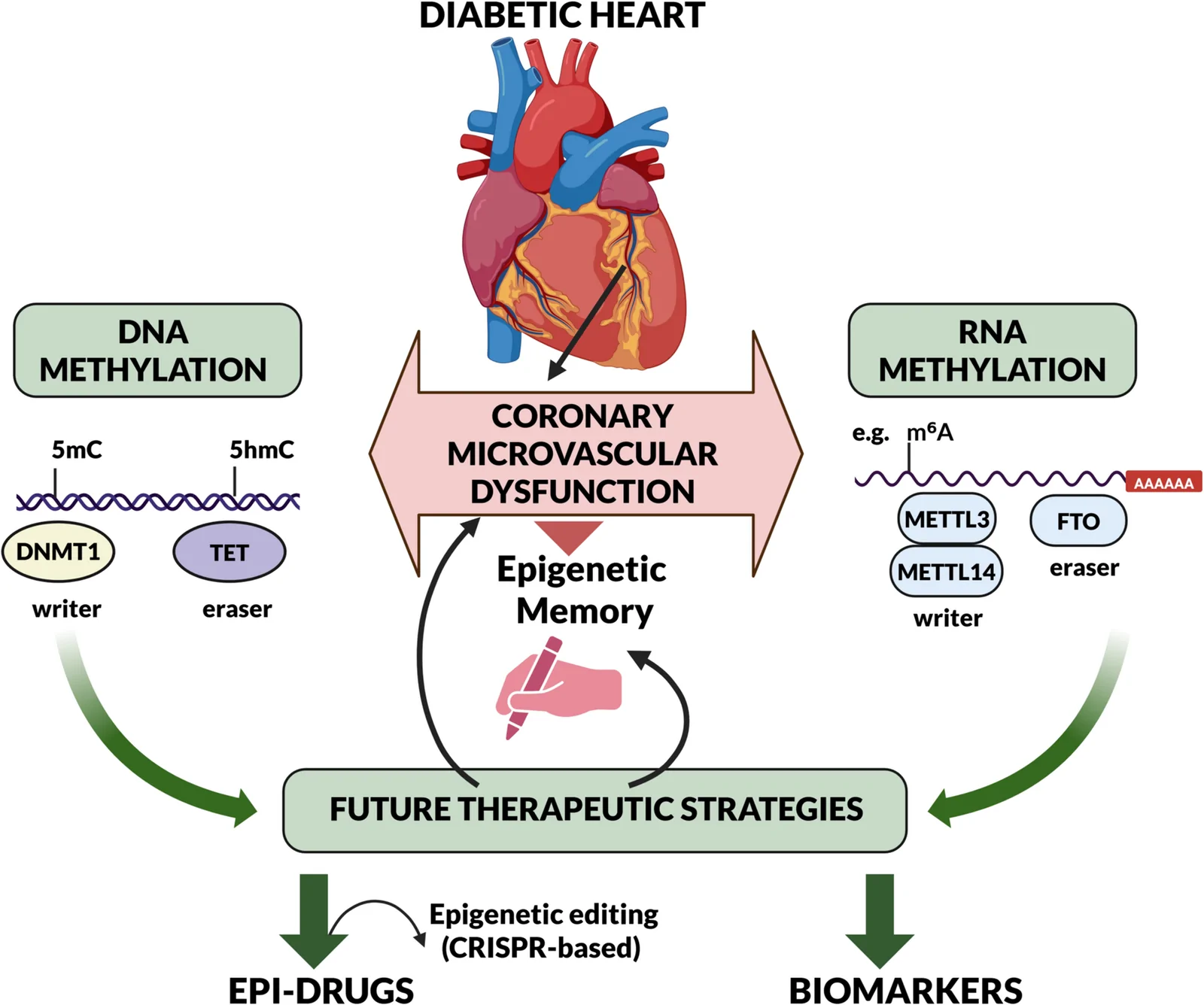
DNA and RNA methylation as potential therapeutic targets for coronary microvascular dysfunction linked with diabetes. Metabolic stress drives coronary microvascular dysfunction (CMD) which is influenced by maladaptive alterations in DNA methylation (5mC) and RNA methylation (e.g. m^6^A). Although such epigenetic changes have not been specifically linked to CMD, aberrant dysfunction-initiating and disease-driving alterations in DNA and RNA methylation are reported in multiple Endothelial Cell types, as evidence of likely pathophysiological importance. Both modifications have emerged as strong therapeutic candidates, with their established contribution to disease memory (even following optimised treatment) gaining particular attention. DNA methylation is regulated by DNMT1 (‘writer’) and TET enzymes (eraser), which control levels of 5mC and 5-hydroxymethylcytosine (5hmC) to modulate gene expression. RNA methylation is mediated by METTL3/METTL14 (‘writers’) and FTO (‘eraser’), governing mRNA stability, translation, and splicing. Selective targeting of these epigenetic pathways represents a promising strategy for CMD, such as development of novel biomarkers and epigenetic drugs (epi-drugs), with CRISPR-based epigenetic editing offering exciting potential for precision intervention

In conclusion, epigenetic signalling is emerging as a central mechanism driving development and progression of CMD in diabetes which offers clear promise as an innovative target for therapeutic intervention and clinical management, as summarised in Fig. 2. It is critical to precisely understand the role of DNA and RNA methylation, as the most common epigenetic modifications, in coronary microvascular ECs towards design of translational strategies which can reverse dysregulated signalling caused by cardiometabolic stress. Such targeted interventions hold exciting potential to protect EC homeostasis whilst altering the trajectory of CMD and adverse cardiac remodelling rather than simply alleviating symptoms as the major focus of current clinical management. Epigenetic-based biomarkers can play a complementary role by capturing systemic metabolic, inflammatory, and vascular stress profiles, to support early identification of high-risk individuals, monitor therapeutic responses, and guide development of personalised disease-modifying therapies. Taken together, these exciting translational research directions position epigenetic targeting as an innovative mechanistically informed precision medicine strategy for CMD which addresses a central early pathogenic driver of heart failure in diabetes.

## Data Availability

No datasets were generated or analysed during the current study.

## Abbreviations

5-aza-Dc: 5-aza-2′-deoxycytidine (decitabine)
ABR: Active BCR-related gene
Akt: Protein kinase B
AML: Acute myeloid leukaemia
ApoE⁻/⁻: Apolipoprotein E knockout
CDK6: Cyclin-dependent kinase 6
CMD: Coronary microvascular dysfunction
CpG: Cytosine-phosphate-guanine
CRISPR: Clustered Regularly Interspaced Short Palindromic Repeats
CysC: Cystatin C
DNMT1: DNA methyltransferase 1
DNMT3B: DNA methyltransferase 3B
EC: Endothelial cell
EFEMP1: EGF-containing fibulin extracellular matrix protein 1
EndMT: Endothelial-to-mesenchymal transition
EpiScore: Epigenetic risk score
FTO: Fat mass and obesity-associated protein
Foxp3: Forkhead box P3
GSTT1: Glutathione S-transferase theta 1
HNRNPC: Heterogeneous nuclear ribonucleoprotein C
HOXA9: Homeobox A9
HUVEC: Human umbilical vein endothelial cell
ICAM-1: Intercellular adhesion molecule 1
IGF2: Insulin-like growth factor 2
IL-1β: Interleukin-1 beta
IL-6: Interleukin-6
Ldlr⁻/⁻: Low-density lipoprotein receptor knockout
LPS: Lipopolysaccharide
LXRα: Liver X receptor alpha
m⁶A: N6-methyladenosine
MACE: Major adverse cardiovascular events
MEG3: Maternally expressed gene 3
METTL3: Methyltransferase-like 3
METTL14: Methyltransferase-like 14
MnSOD: Manganese superoxide dismutase
mRNA: Messenger RNA
mTOR: Mechanistic target of rapamycin
NF-κB: Nuclear factor kappa B
NO: Nitric oxide
PI3K: Phosphoinositide 3-kinase
PLAU: Plasminogen activator, urokinase
PPARγ1: Peroxisome proliferator-activated receptor gamma 1
PSEN1: Presenilin 1
PTX3: Pentraxin 3
RG108: 2-(1,3-dioxoisoindolin-2-yl)-3-(1 H-indol-3-yl) propanoic acid
ROBO4: Roundabout guidance receptor 4
RNA: Ribonucleic acid
SIRT6: Sirtuin 6
SMOC2: SPARC-related modular calcium-binding protein 2
STAT3: Signal transducer and activator of transcription 3
TET: Ten-eleven translocation
TNF-α: Tumour necrosis factor alpha
TNIP1: TNFAIP3-interacting protein 1
Tregs: Regulatory T cells
TSDR: Treg-specific demethylated region
TSP-1: Thrombospondin 1
YTHDC1: YTH domain-containing protein 1
